# A hydrodynamic interaction between bubbles and gas supply system during gas bubble departures in liquids: an experimental study

**DOI:** 10.1038/s41598-023-45362-2

**Published:** 2023-10-20

**Authors:** P. Dzienis, K. Golak, M. Konopka, R. Mosdorf, K. Bazienė, J. Gargasas

**Affiliations:** 1 Faculty of Mechanical Engineering, Bialystok University of Technology, Wiejska 45C, 15-351 Białystok, Poland; 2 Faculty of Mechanics, Vilnius Gediminas Technical University, Saulėtekio al. 11, 10223 Vilnius, Lithuania

**Keywords:** Ocean sciences, Fluid dynamics

## Abstract

In the present paper, the hydrodynamic interactions between bubbles and the gas supply system to a needle were experimentally investigated. In experimental investigations in one of the needles, the air volume flow rate was constant, and in the neighbouring needle, it was changed. In the paper, the methods of data analysis: wavelet decomposition, and FFT were used. It was shown that the hydrodynamic interaction becomes stronger with the increase in air volume flow rate supply to the needle. The occurrence of hydrodynamic interaction modifies bubble growth time slightly, but it significantly modifies the bubble waiting time. In the case when the liquid penetration into the needle is repeatable, then the percentage disturbances in bubble growth time and bubble waiting time are close to each other. Moreover, it can be concluded that synchronized or alternative bubble departures from twin neighbouring needles (occurring due to hydrodynamic interaction) are possible by modifying the bubble waiting time. The modification of hydrodynamic interaction between bubbles, the bubbles themselves, and gas supply systems can be used to control the bubble departure process.

## Introduction

Understanding the bubble departure process in liquids is necessary to better control the aeration or saturation process, which helps e.g. in purifying surface water or municipal sewage. Control of the bubble departure process can be used to intensify mass transfer in the bubble column^1–3^. The interaction between bubbles is important in investigations of the bubble formation process during boiling^4–6^. Moreover, investigations of bubble departure, interactions between bubbles and bubble coalescence are treated as an introduction to investigations of the greenhouse effect caused by escaping methane bubbles in oceans^7,8^.

There are a lot of papers in which the results of experiments of bubble generation from a single needle^2,9–15^ and twin or more needles or orifices^15–22^ are described. In papers^9,10,23^ the process of liquid penetration into the needle during bubble departures was analysed. The bubble departure time can be divided into bubble growing time and bubble waiting time^9,11,23^. During the bubble waiting time, for relatively small gas volume flow rates, the needle or orifice is penetrated by the liquid. This is caused by an increase in gas pressure in the gas supply system^9,11^. The investigations of liquid penetration into the needle or orifice were analysed only for bubbles departing from a single needle.

In papers^10,12,13^, the chaotic nature of the bubble departure process and bubble trajectories were investigated. In^10^, it was shown that the chaotic character of bubble trajectories is caused by the departed bubble’s shape and liquid flow generated by the moving bubble in the bubble column. Moreover, two groups of phenomena responsible for the chaotic nature of bubble behaviours (the first group—bubble interface oscillations, liquid flow around the needle, and the second group—processes which appear in the gas supply system) are proposed^12–14^. It can be assumed that the liquid flow above the needle can modify the processes that appear in the needle, and consequently, the pressure fluctuation in the gas supply system.

The influence of the distance between needles on the interaction between bubbles departing from them was investigated in papers^15,16^. It was shown that the hydrodynamic interaction can lead to chaotic pressure changes in the gas supply system and consequently chaotic bubble departures^15^. The regimes based on synchronization among orifices and the parameters affecting the bubbling dynamics were described in^16^. In the paper^15^, the coefficient of alternative bubble departure from twin needles (*ABD*) was proposed. It was shown that the interaction between bubbles depends on the distance between needles, air volume flow rate, liquid properties, and bubble departure frequencies. Additionally, the *ABD* coefficient was analysed for orifices in the paper^18^. Moreover, the interaction between bubbles was investigated in papers^19–21^, and it was shown that this interaction modifies the bubble’s trajectories. In the paper^19^, bubbles were generated in the water and an aqueous glycerine solution. It was concluded that bubble interactions and bubble coalescence depend on gas flow rates, the distance between tubes, and liquid properties. In^20–22^, it was shown that interactions between bubbles can lead to bubble coalescence or bubble bouncing. The manner of bubble interaction depends on the bubble Reynolds number. Bubble coalescence is investigated for bubbles growing from nucleation sites too^24^. In paper^24^, it was shown that bubble coalescence depends on the contact angle because it modifies the diameter of the growing bubble.

The hydrodynamic interaction between bubbles was investigated only for bubbles departing from two or more needles, but the influence of liquid flow caused by departing bubbles on pressure fluctuation in the gas supply system, and consequently, liquid penetration into the needle, has not been investigated yet. Additionally, there is no research in which the influence of hydrodynamic interaction on bubble growth time and waiting time was investigated. If the interaction between the bubble and the gas supply system modifies the bubble waiting time, then it can be assumed that modification of the liquid flow above the needle can be used to control the bubble departure process. Preventing the bubble coalescence during their growth or their departures using the hydrodynamic interaction is important to control the release of methane in oceans or increase the efficiency of aeration during water purification.

In the present paper, the influence of hydrodynamic interaction—liquid flow above the needle, modified by departed bubbles, was experimentally investigated. In the experiment, bubbles were generated from two glass needles. Based on the results presented in the paper^16^, the distance between needles was set as 4 mm, and air volume flow rates were set in one of the needles in the range of 0.00492–0.0424 l/min. For that selected distance between needles and air volume flow rates, the hydrodynamic interaction between bubbles should occur. In order to identify the occurrence of hydrodynamic interaction, wavelet decomposition and FFT data analysis methods were used. To identify the influence of hydrodynamic interaction on the disturbances in the gas supply system (based on liquid penetration into the needle), 3D attractors were reconstructed. The bubble growth time and waiting time were estimated. The structure of the paper is as follows. In the “Experimental setup and data characteristics” section of this paper, the experimental setup and data characteristics are described. Results of the experimental data analysis and discussion are shown in the “Results of experimental investigations” section. A summary of the obtained results is shown in the “Conclusion” section.

## Experimental setup and data characteristics

**Figure 1:**
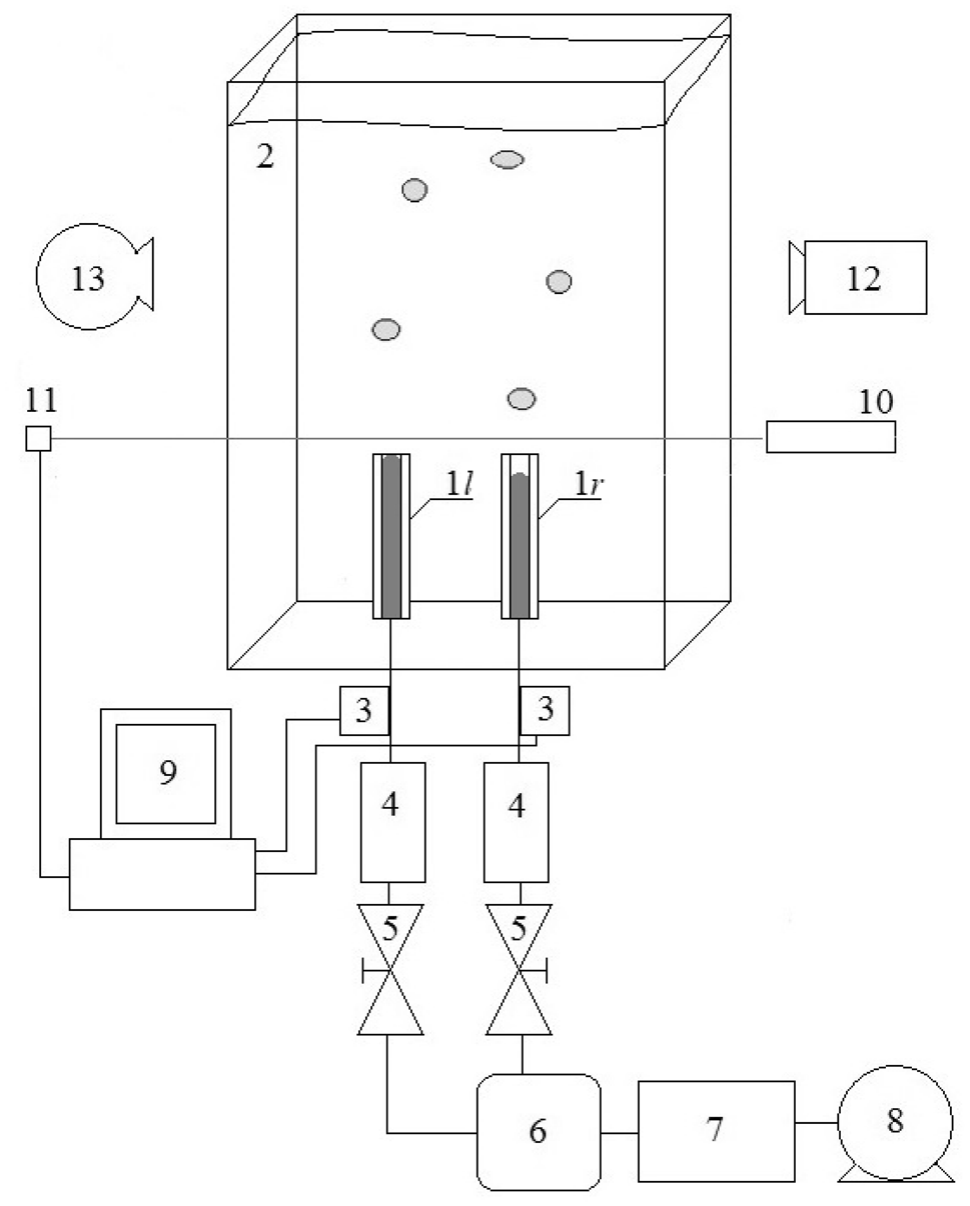
Diagram of the experimental setup. 1—glass needles (1* l*—left needle, 1*r*—right needle), 2 –glass tank, 3—pressure sensors, 4—flowmeters, 5—air valves, 6—air tank, 7—pressure regulator, 8—air pump, 9—computer acquisition system, 10—laser, 11—phototransistor, 12—high-speed camera, 13—light source.

In the experimental setup, bubbles were generated into water from twin glass needles placed at the bottom of the tank. The inner diameters of the needles were equal to 1 mm, and the distance between needle outlets was 4 mm. The lengths of the needles were equal to 75 mm. The transverse diameters of departed bubbles (parallel to the edge of the needle) were changed in the range of 3.2–3.8 mm (the measurement error was equal to 1 pixel and it is about 0.07 mm). The tank dimensions were equal to 300 × 150 × 700 mm (length  ×  width  ×  height). In the experiment, the water temperature was controlled by a MAXIM DS18B20 digital thermometer (with an accuracy of 0.1 °C) and was equal to 20 °C. A diagram of the experiment is shown in Fig. 1.

In order to investigate interactions between bubbles and the gas supply system in one of the needles (the right needle), the air volume flow rate was constant, and in the second one (the left needle), it changed during the experiment. In the right needle, the air volume flow rate was set using the air valve at the value of 0.00632 l/min. For that selected air volume flow rate, liquid penetration into the needle was observed. When the gas was not supplied to the left needle, then the depth of liquid penetration into the right needle was equal to 6 mm (the measurement error was equal to 1 pixel and it is about 0.07 mm). In the left needle, the air volume flow rate was changed during the measurements, in the range of 0.00492–0.0424 l/min. The air volume flow rates were measured using flow meters—BROOKS Sho-Rate Purgemeter Model GT 1355 with an accuracy ± 2%. The repeatability of measurements was equal to ± 0.5%. Flow meters were connected to an air tank, which was powered by an air pump. The pressure in the air tank was controlled by a Metalwork Regtronic proportional pressure-reducing valve. During the experimental investigation, the air pressure was set to 0.03 MPa, with an accuracy equal to 0.5%. The proportional pressure-reducing valve was used to ensure constant pressure conditions during the bubble departures and to eliminate pneumatic interaction between the needles. Moreover, in order to eliminate pneumatic interaction between the needles the lengths of the gas supply lines between the pressure tank and the nozzle were appropriately selected. The length of them was equal to 1.7 m. The gas lines were made from brass tubes with an inner diameter equal to 2 mm. For that selected dimensions pressure drops in the gas supply lines were higher than the pneumatic interaction between the needles.

**Figure 2:**
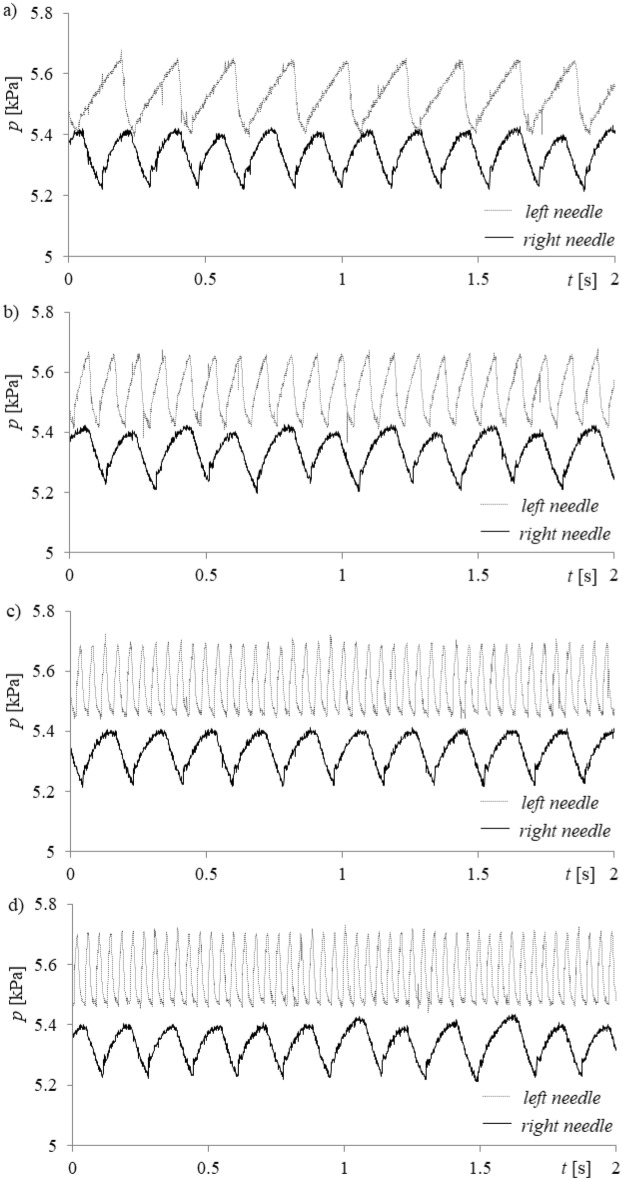
Examples of recorded time series of pressure changes in the gas supply system for constant air volume flow rate in the right needle *q*_*r*_ = 0.00632 l/min and selected air volume flow rates in the left needle *q*_*l*_. (**a**) *q*_*l*_ = 0.00492 l/min, (**b**) *q*_*l*_ = 0.0127 l/min, (**c**) *q*_*l*_ = 0.0334 l/min, (**d**) *q*_*l*_ = 0.0424 l/min.

During the experiment, the time series of air pressure fluctuations in both needles and videos of liquid movement inside the glass needles were recorded simultaneously. Air pressure fluctuations were measured with the use of a Freescale Semiconductor MPX12DP silicon pressure sensor (sensitivity was 5.5 mV/kPa). The pressure sensor response time was equal to 1 ms. Time series of the air pressure changes were recorded by a Data Translation DT9804 data acquisition system. The sampling frequency was equal to 1 kHz. An example time series of pressure changes for selected air volume flow rates in the left needle and air volume flow rate equal to 0.00632 l/min in the right needle are shown in Fig. 2.

The mean pressure in the left needle was kept slightly higher than in the right needle (Fig. 2). This was done by reducing the cross-section of the gas supply line to the left needle by 2% with respect to the cross-section of the supply line to the right needle. In order to reduce the cross-section, the brass tube was bent it enters the needle. For bent a pipe crimper was used. Its diameter was measured using a digital microscope VHX-7000 with a zoom of 1000×. Based on the diameter the cross-section was calculated and compared with the cross-section value of the right needle supply pipeline. This was done to set the left needle as the needle controlling the bubble departure process. In addition, the increase in gas pressure in the supply system leads to a more orderly bubble departure process.

**Figure 3:**
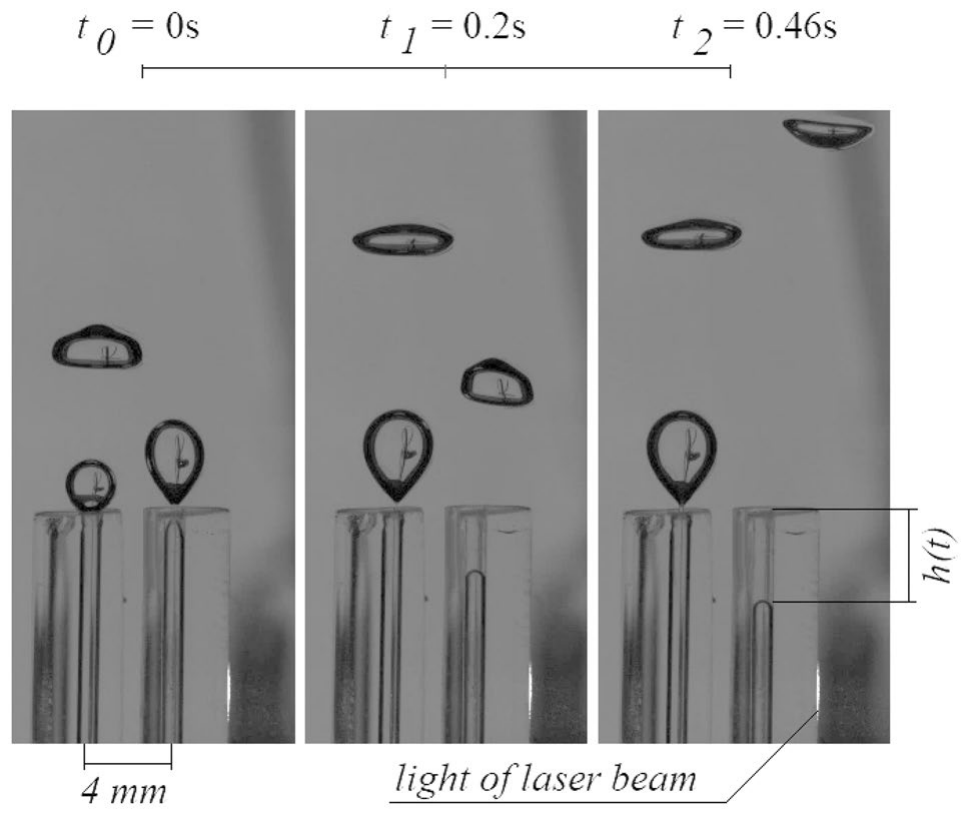
Example frames of films for air volume flow rate supplied to the left needle *q*_*l*_ = 0.0424 l/min and air volume flow rate equal supplied to the right needle *q*_*r*_ = 0.00632 l/min.

The liquid penetration into the needle was recorded with a Phantom v1610 high-speed camera. Videos were recorded in grayscale. The framerate of the recording was equal to 5000 fps. The resolution of the videos was 384 × 640 pixels. 1 pixel in the frame was about 0.07 mm. The durations of the videos were equal to 10 s. Videos were divided into frames. Example frames of films are shown in Fig. 3.

**Figure 4:**
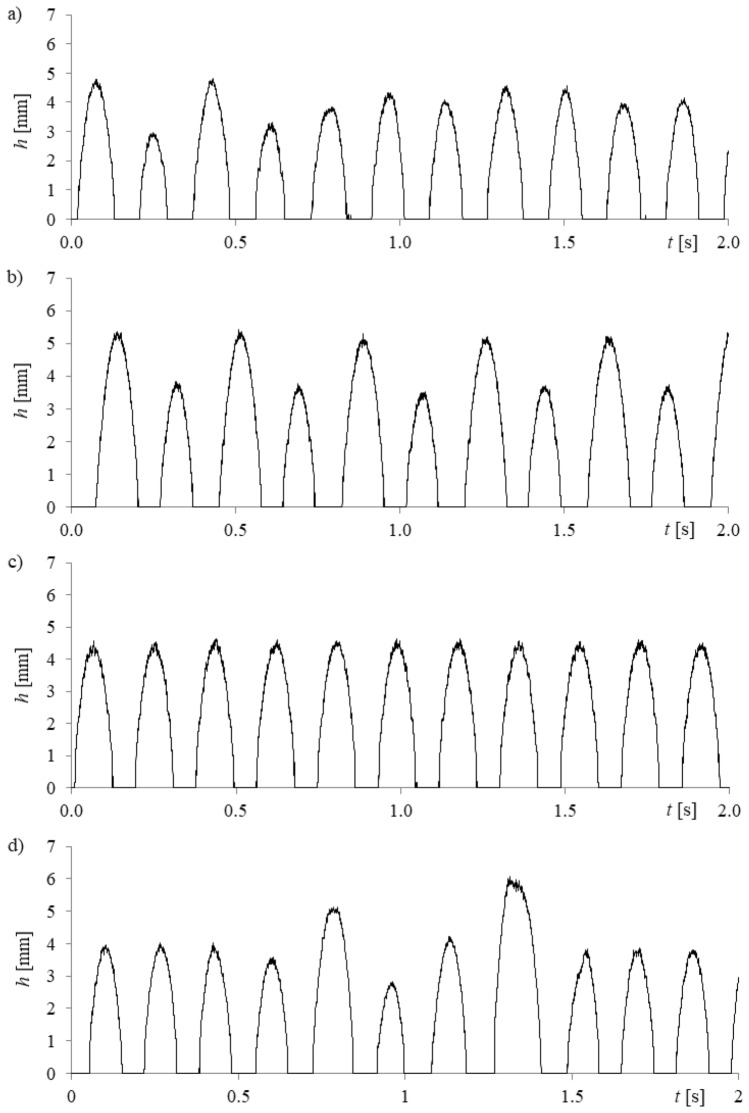
Examples of time series of liquid penetration into the right needle for constant air volume flow rate in the right needle *q*_*r*_ = 0.00632 l/min and selected air volume flow rates in the left needle *q*_*l*_. (**a**) *q*_*l*_ = 0.00492 l/min, (**b**) *q*_*l*_ = 0.0127 l/min, (**c**) *q*_*l*_ = 0.0334 l/min, (**d**) *q*_*l*_ = 0.0424 l/min.

Based on the frames (Fig. 3), the time series of liquid penetration into the needle was reconstructed using a self-made computer program. Data from the high-speed camera and data from the acquisition station were synchronized using the laser—phototransistor system. In order to assign the frames containing subsequent bubble stages above the needle to the time series of pressure fluctuations, the time series of pressure fluctuations were resampled to 5000 Hz using a computer program. The synchronization error is related to the frequency of recording by the data acquisition station (1000 Hz) and it is about 0.001 s. The method of data synchronization has been described in the paper^25^. An example time series of liquid penetration into the right needle is shown in Fig. 4.

In Fig. 4, it is shown that an increase in the air flow rate to the left needle modifies the depth and behaviour of liquid penetration into the right needle (the air volume flow rate is constant). It can be assumed that the changes of the frequency of bubble departures from the left needle modify liquid flow (hydrodynamic interaction) above the right needle, and consequently, the bubble growth time and bubble waiting time for bubbles departing from the right needle change. These changes are more visible when analysing the time series of liquid penetration into the needle compared to the time series of pressure fluctuations in the needle.

## Results of experimental investigations

### Analysis of experimental data

**Figure 5:**
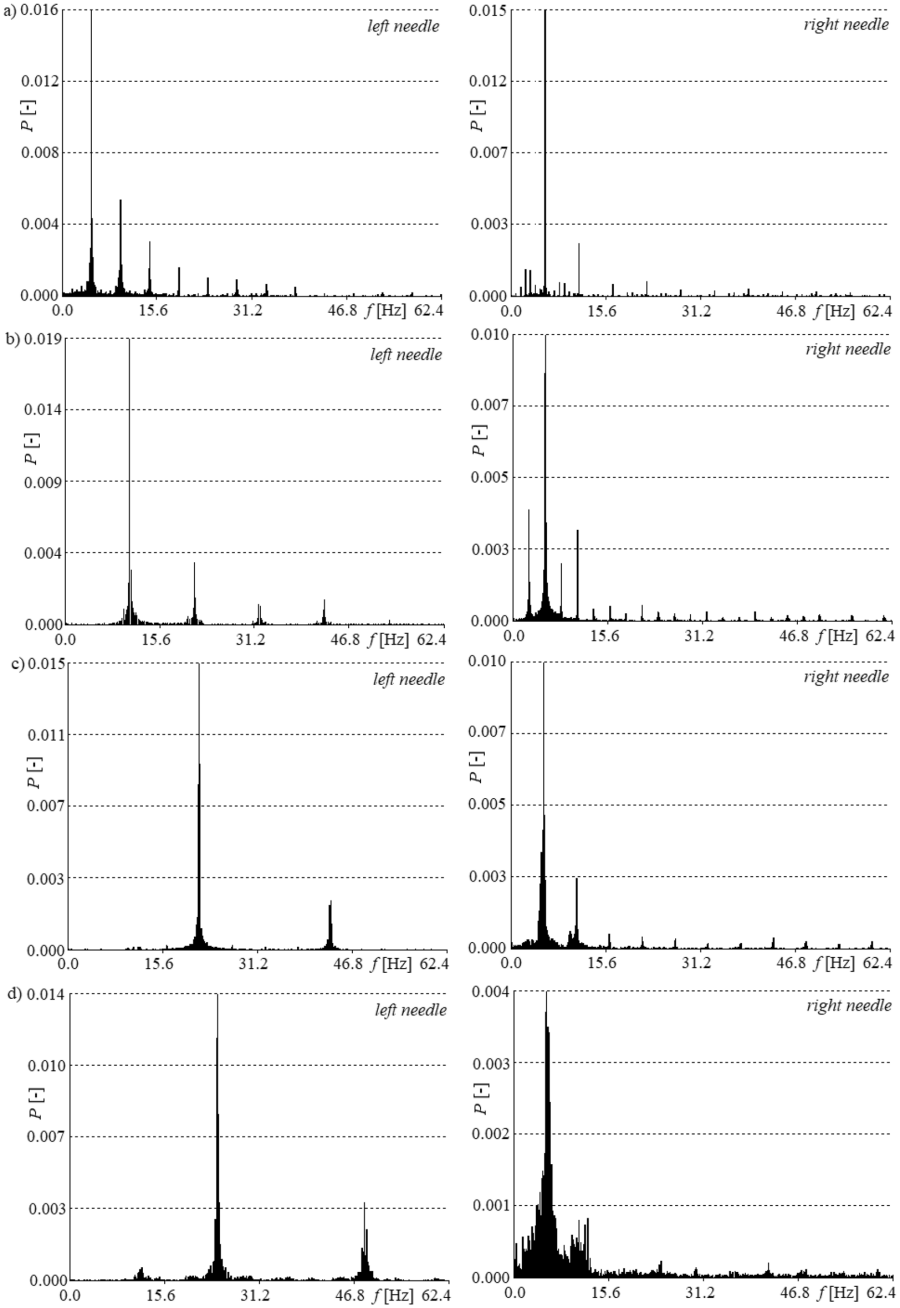
Examples of frequency power spectra of time series of pressure changes in gas supply systems of left and right needles, for constant air volume flow rate in the right needle *q*_*r*_ = 0.00632 l/min and selected air volume flow rates in the left needle *q*_*l*_. (**a**) *q*_*l*_ = 0.00492 l/min, (**b**) *q*_*l*_ = 0.0127 l/min, (**c**) *q*_*l*_ = 0.0334 l/min, (**d**) *q*_*l*_ = 0.0424 l/min.

In order to check the time shift of bubble departures from both needles, the frequencies of bubble departures were estimated. The frequency of bubble departure from the left and right needle was estimated based on the time series of pressure changes in the gas supply system. For this purpose, the FFT method^26,27^ was used, and the examples of power spectra are shown in Fig. 5.

The dominant frequency of pressure changes in the left needle can be treated as a frequency of bubble departures (Fig. 5). In the case of pressure fluctuations in the right needle, the dominant frequency is treated as the departure frequency of most bubbles in the analysed time series. The hydrodynamic interaction modifies the frequency of bubble departure, and consequently, it can be different for subsequent cycles of bubble departures. It can be assumed that the hydrodynamic interaction can modify bubble waiting and bubble growing time.

In Fig. 5, it was shown that the frequency of bubble departures from the left needle is similar in subsequent cycles of bubble departures (frequencies are close to the dominant frequency in the graphs on the left side of Fig. 5). In the power spectra received from the pressure changes in the right needle, we can distinguish a few ‘dominant’ frequencies (on the right side of Fig. 5). This means that bubbles depart with different frequencies, and it suggests that the bubble departure frequency from the right needle adapts to the bubble departure frequency from the left needle. The right needle can be treated as a slave needle and the left needle can be treated as a master needle. The process of bubble departures from the left needle controls bubble departures from the right needle.

The dominant frequency of the time series of pressure fluctuations in the left (*f*_*l*_) and the “greatest” dominant frequency in the right (*f*_*r*_) needle are shown in Table 1. These values are shown for selected air volume flow rates in the left needle (*q*_*l*_) and constant air volume flow rate in the right needle (*q*_*r*_ = 0.00632 l/min). Moreover, Table 1 shows the ratio of *f*_*l*_ to *f*_*r*_.

**Table 1:** Dominant frequency of time series of pressure fluctuations in left (*f*_*l*_) and right (*f*_*r*_) needles for selected air volume flow rates in the left needle (*q*_*l*_) and constant air volume flow rate in the right needle (*q*_*r*_ = 0.00632 l/min).

*q*_*l*_ (l/min)	0.00492	0.0127	0.0334	0.0424
*f*_*l*_ (Hz)	4.8	10.4	21.6	24.3
*f*_*r*_ (Hz)	5.7	5.5	5.4	5.7
*f*_*l*_* / f*_*r*_ (–)	0.84	1.89	4.00	4.26

The dominant frequency cannot be treated as the bubble departure frequency, but analysis of the dominant frequency and FFT power spectra can be used to determine the adjustment of the bubble departures from the right needle to the bubbles departing from the left needle. Adjustment of the bubble departures was observed for an air volume flow rate in the left needle equal to *q*_*l*_ = 0.0334 l/min, in which case the ratio of *f*_*l*_* / f*_*r*_ was equal to 4.

In order to determine the repeatability of liquid penetration into the needle and pressure changes in gas supply systems to the needles, 3D attractors were reconstructed. The attractor reconstruction was carried out using the stroboscope coordination. This method calculates subsequent coordinates of attractor points based on the samples between which the distance is equal to the time delay. If the subsequent trajectories in the attractor are close to each other, then the signal is quasi-periodic. If the subsequent trajectories on the attractor reconstruction start to diverge from each other, it means the analyzed signal is chaotic. During the reconstruction of the 3D attractor, the time delay (*τ*) was estimated for all time series separately. To determine *τ,* the mutual information method was used^28–30^. In this method, the first minimum of the following function is treated as the proper value of *τ*:

1 $$ I\left( {x_{i} ,x_{i + \tau } } \right) = \sum\limits_{{x_{i + \tau } }} {\sum\limits_{{x_{i} }} {p\left[ {x_{i} ,x_{i + \tau } } \right]} } \log_{2} \left\{ {\frac{{p\left[ {x_{i} ,x_{i + \tau } } \right]}}{{p\left[ {x_{i} } \right],p\left[ {x_{i + \tau } } \right]}}} \right\}, $$

where: $p\left[ {x_{i} ,x_{i + \tau } } \right]$ is the joint probability function of $\{ x_{i} \}$ and $\left\{ {x_{i + \tau } } \right\}$, $p\left[ {x_{i} } \right]$ and which are the marginal probability distribution functions of $\{ x_{i} \}$ and $\left\{ {x_{i + \tau } } \right\}$.

The values of *τ* for time series of pressure fluctuations in the left and right needles and liquid penetration into the right needle are shown in Table 2. The values of *τ* are shown for selected air volume flow rates in the left needle (*q*_*l*_) and constant air volume flow rate in the right needle (*q*_*r*_ = 0.00632 l/min).

**Table 2:** Values of time delay for time series: pressure fluctuations in the left needle (*τ*_*pl*_), pressure fluctuations in the right needle (*τ*_*pr*_) and liquid penetration into the needle (*τ*_*lp*_) for selected air volume flow rates in the left needle (*q*_*l*_) and constant air volume flow rate in the right needle (*q*_*r*_ = 0.00632 l/min).

*q*_*l*_ [l/min]	0.00492	0.0127	0.0334	0.0424
*τ*_*pl*_ [-]	27	15	8	5
*τ*_*pr*_ [-]	30	34	30	30
*τ*_*lp*_ [-]	140	157	147	142

**Figure 6:**
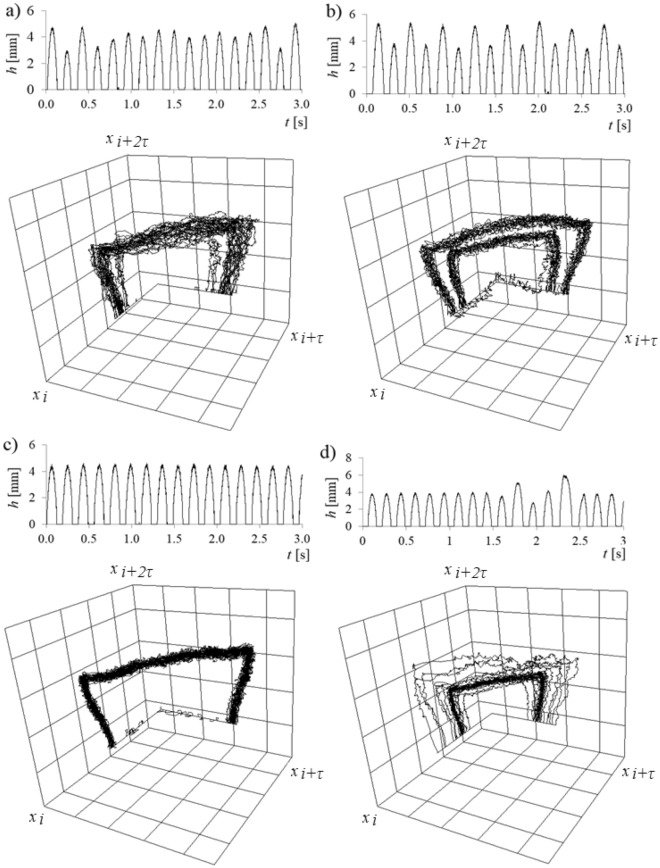
3D attractor reconstructions of time series of liquid penetration into the right needle for constant air volume flow rate in the right needle *q*_*r*_ = 0.00632 l/min and selected air volume flow rates in the left needle *q*_*l*_. (**a**) *q*_*l*_ = 0.00492 l/min, (**b**) *q*_*l*_ = 0.0127 l/min, (**c**) *q*_*l*_ = 0.0334 l/min, (**d**) *q*_*l*_ = 0.0424 l/min.

Examples of time series of liquid penetration into the needle and 3D attractor are shown in Fig. 6.

In Fig. 6, it is shown that the increase in air volume flow rate (and bubble departure frequency) in the left needle modifies the nature of liquid penetration into the right needle. In Fig. 6a, it is shown that for air volume flow rate in the left needle *q*_*l*_ = 0.00492 l/min, liquid penetration into the right needle fluctuated unpredictably. The trajectories forming 3D attractors are not repeatable. In Fig. 6b, the depth of liquid penetration into the needle changes with two characteristic periods—in this case, the air volume flow rate in the left needle *q*_*l*_ = 0.0127 l/min. The first depth of liquid penetration is close to 5.2 mm and the second one is close to 4 mm. We can distinguish two overlapping trajectories on the 3D reconstruction of the attractor. In Fig. 6c, one sequence of liquid penetration was observed, and the depth of liquid penetration into the needle was close to 4.5 mm. The air volume flow rate in the left needle was *q*_*l*_ = 0.0334 l/min. In this case, the trajectories forming the 3D attractor are repeatable. In Fig. 6d, the period of repeatable values of depth of liquid penetration into the needle (for 9 subsequently departed bubbles), and after that, the disappearance of the repeatable depth of liquid penetration was observed. After the departure of 4 bubbles, the appearance of repeatable liquid movements was observed. In the 3D attractor reconstruction (Fig. 6d), we can see repetitive trajectories (thicker trajectory) and the appearance of no repeatable changes of liquid penetration depth (lines away from the thicker trajectory). *τ*

**Figure 7:**
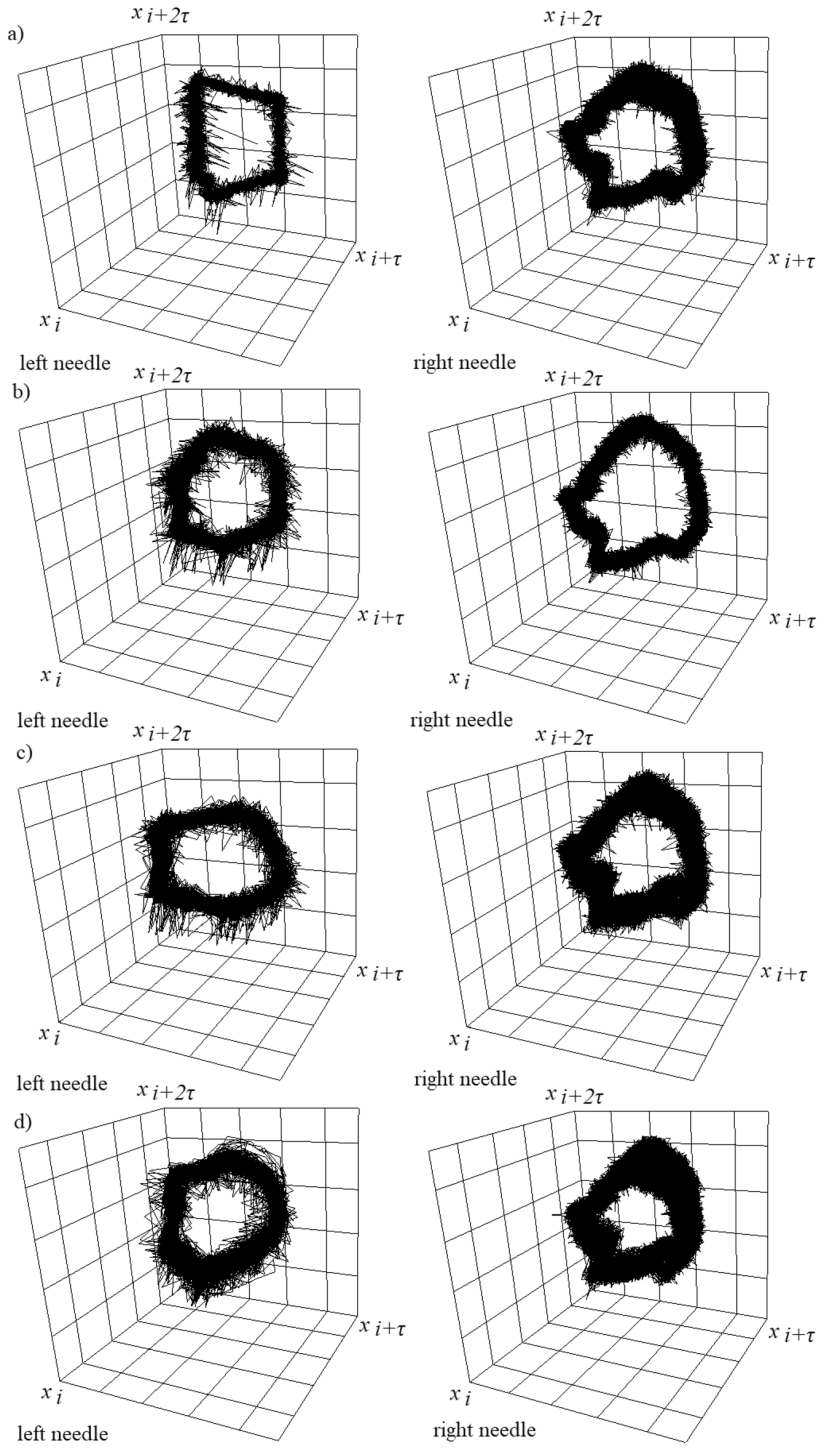
3D attractor reconstructions of time series of pressure fluctuations in the left and right needle for constant air volume flow rate in the right needle *q*_*r*_ = 0.00632 l/min and selected air volume flow rates in the left needle *q*_*l*_. (**a**) *q*_*l*_ = 0.00492 l/min, (**b**) *q*_*l*_ = 0.0127 l/min, (**c**) *q*_*l*_ = 0.0334 l/min, (**d**) *q*_*l*_ = 0.0424 l/min.

Examples of 3D attractors of air pressure changes in the left and right needle are shown in Fig. 7.

The trajectories forming the 3D attractors (Fig. 7) obtained from a time series of pressure fluctuations in the left needle are closer to each other than the trajectories in the 3D attractors obtained from a time series of pressure fluctuations in the left needle. The closer the trajectories on the 3D attractor, the more orderly the investigated process is. This confirms that the bubble departures from the left needle are the processes controlling the bubble departures from the right needle. The most chaotic pressure fluctuations in the right needle are observed for the cases in Fig. 7a,d. In these cases, the liquid penetrations into the needle are not repeatable for subsequent bubble departures. The most orderly pressure fluctuations in the right needle occur for air volume flow rate in the left needle *q*_*l*_ = 0.0127 l/min (Fig. 7b). For this flow rate, we observed periodic liquid penetration into the needle with two characteristic periods (Fig. 6b). In the case presented in Fig. 7c, the pressure fluctuations in the right needle are more chaotic than in the case presented in Fig. 7c, but the liquid penetrations into the needle in subsequent bubble departure cycles are quasi-periodic.

The obtained results are consistent with the results presented in the paper^10^, i.e. pressure fluctuations are more chaotic in comparison with the changes in depth of liquid penetration into the needle, and these changes of pressure fluctuations can lead to periodic bubble departures. In this case, the chaotic changes in pressure fluctuations can lead to synchronized or alternative bubble departures from twin needles and can be used to control the bubble departure process from one of the needles.

**Figure 8:**
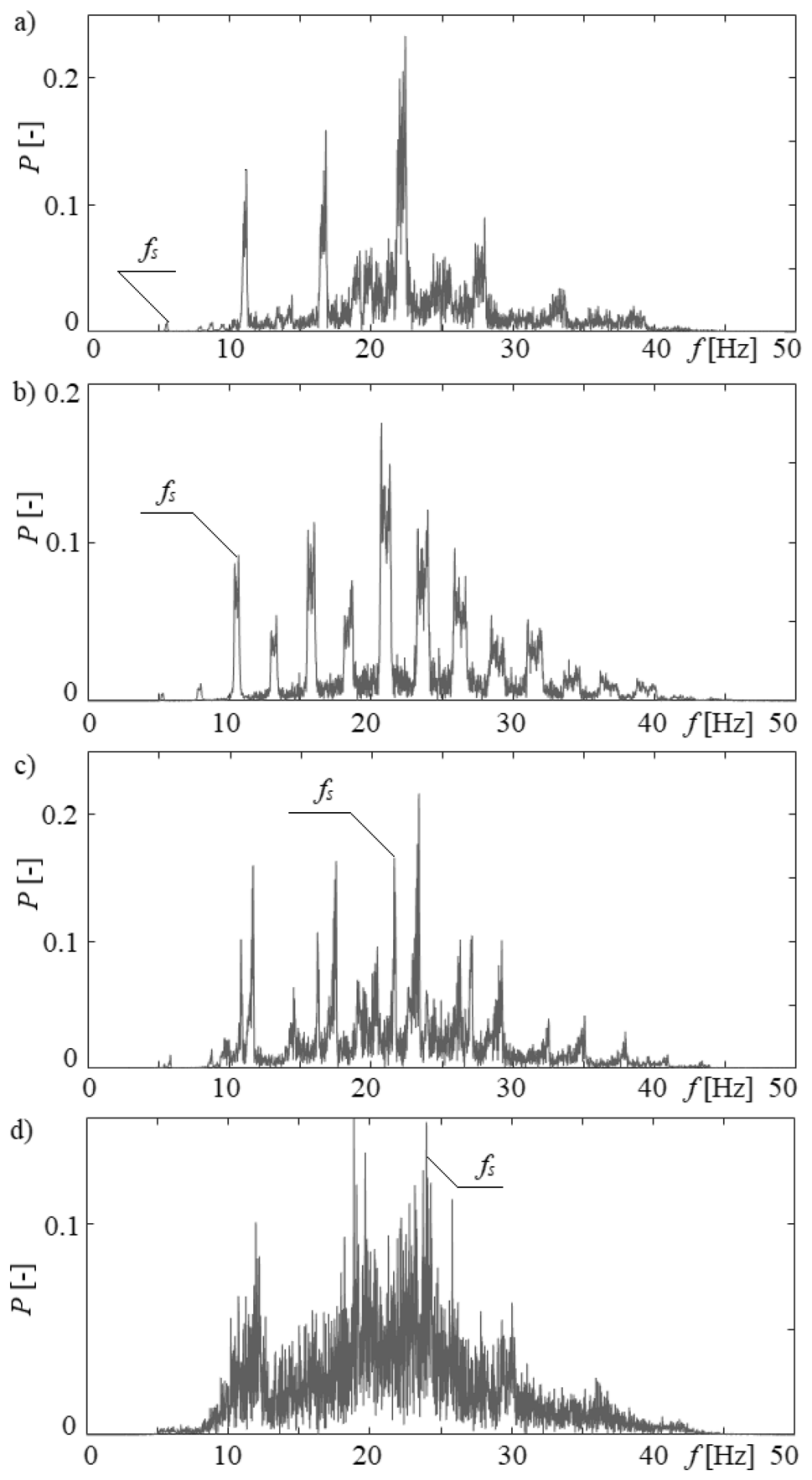
Frequency components obtained from 5th level wavelet decomposition of time series of liquid penetration into the right needle for constant air volume flow rate in the right needle *q*_*r*_ = 0.00632 l/min and selected air volume flow rates in the left needle *q*_*l*_. (**a**) *q*_*l*_ = 0.00492 l/min, (**b**) *q*_*l*_ = 0.0127 l/min, (**c**) *q*_*l*_ = 0.0334 l/min, (**d**) *q*_*l*_ = 0.0424 l/min.

Wavelet decompositions of liquid penetration into the needle were performed in order to check the occurrence of hydrodynamic interaction between bubbles departing from the left needle and liquid penetration into the right needle. This analysis was performed in Matlab with the Wavelet Toolbox. Due to the non-linear nature of the analysed time series, we chose the Daubechies (*db2*) method in the Orthogonal Wavelet Family, and we performed five levels of frequency decomposition. The signal of details obtained from the 5th level of decomposition was analysed using the Fast Fourier Transform. As a consequence, we obtained frequency components in the time series of liquid penetration into the needle and the frequency of bubble departures from the left needle is visible in it. The results of wavelet decomposition analysis are shown in Fig. 8.

In Fig. 8a, the air volume flow rate in the left needle was equal to 0.00492 l/min, and the frequency of bubble departure from this needle was 4.8 Hz. This frequency (*f*_*s*_—Fig. 8) is one of the frequency components in the time series of liquid penetration into the right needle. The power spectrum of this frequency is very slight. This means that the hydrodynamic interaction is minor. For air volume flow rate *q*_*l*_ = 0.0127 l/min, the frequency of bubble departures was 10.4 Hz, and this frequency is visible in Fig. 8b. The power spectrum is higher than for air volume flow rate *q*_*l*_ = 0.00492 l/min, and this means that the increase in air volume flow rate in the left needle at constant air volume flow rate in the right needle enhances the hydrodynamic interaction between bubbles and the gas supply system. Furthermore, the occurrence of bubble departure frequencies from the left needle is observed in the other time series of liquid penetration into the right needle in the presented cases—Fig. 8c,d. The obtained results are consistent with the results presented in paper^16^, i.e. the increase in the air volume flow rate supplied to the needles causes intensification of the hydrodynamic interaction between the departing bubbles.

### Influence of interaction between bubbles and gas supply systems on bubble growth and bubble waiting time

**Figure 9:**
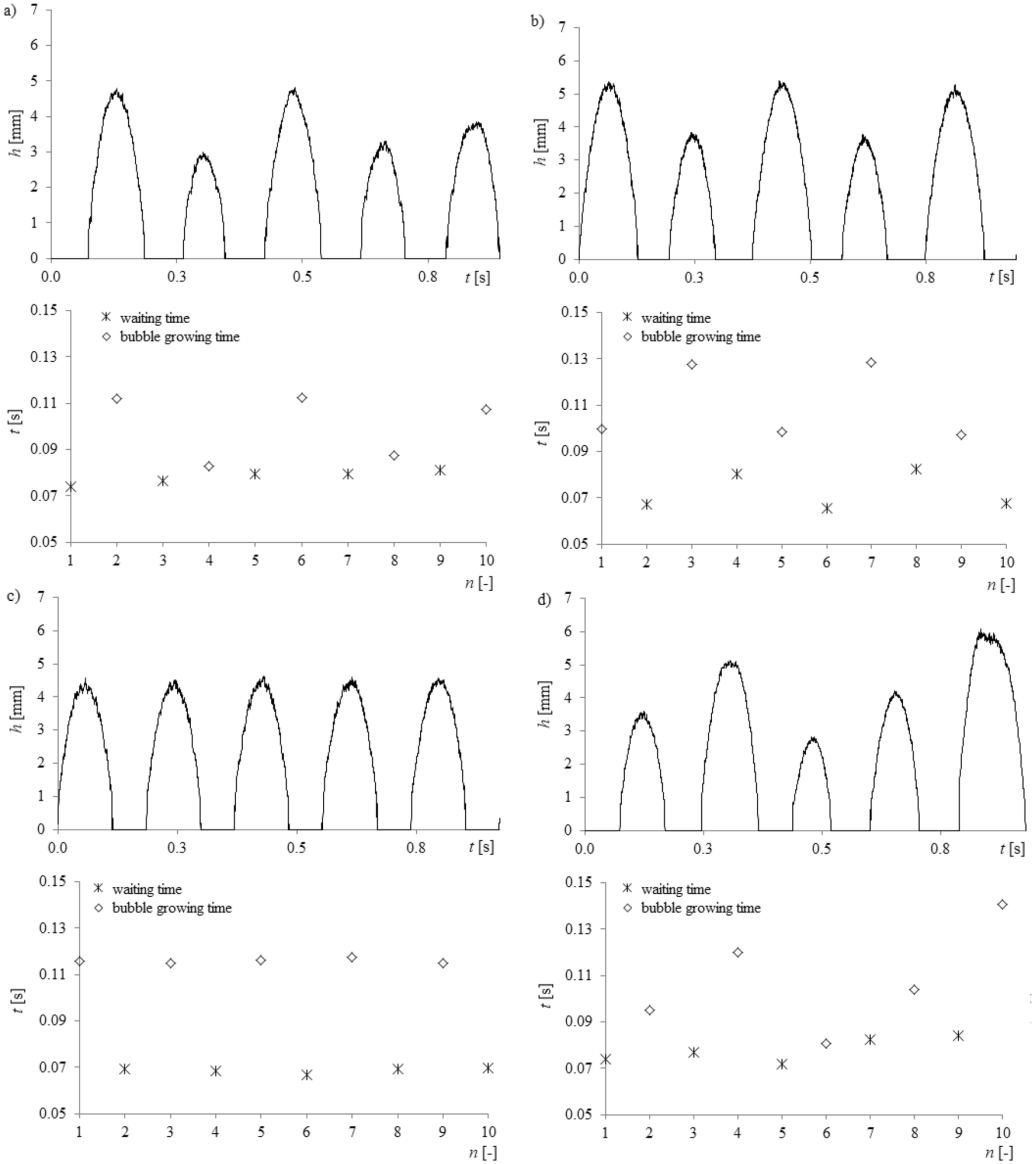
Bubble growth and bubble waiting time for constant air volume flow rate in the right needle *q*_*r*_ = 0.00632 l/min and selected air volume flow rates in the left needle *q*_*l*_. (**a**) *q*_*l*_ = 0.00492 l/min, (**b**) *q*_*l*_ = 0.0127 l/min, (**c**) *q*_*l*_ = 0.0334 l/min, (**d**) *q*_*l*_ = 0.0424 l/min.

The fluctuations of bubble departure frequency are coupled with the perturbation in the bubble waiting and the bubble growth time. Therefore, based on the time series of liquid movement into the needle, the bubble waiting time and bubble growth time were estimated. The bubble growth time (the time in which the depth of liquid penetration into the needle *h*(*t*) = 0) and the bubble waiting time (h(t) ≠ 0)) are shown in Fig. 9 for 5 subsequent bubbles.

**Figure 10:**
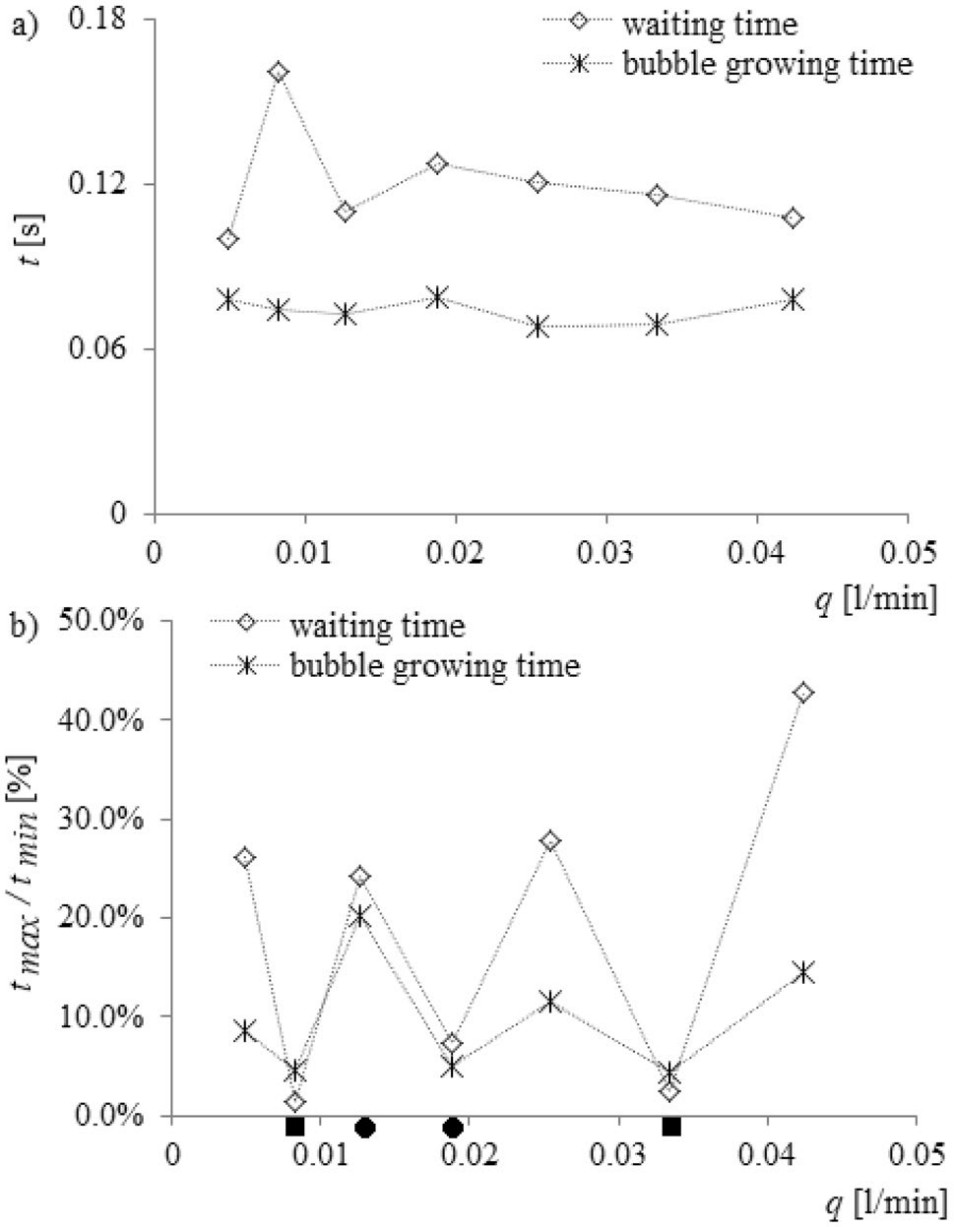
Mean values of bubble growth time, waiting time, their percentage changes vs. changing air volume flow rate in the left needle and for constant air volume flow rate in the right needle *q*_*r*_ = 0.00632 l/min (**a**) mean values of bubble growth time and waiting time, (**b**) percentage changes of bubble growth time and waiting time.

In Fig. 9, bubble waiting time was marked using asterisks and the bubble growth time was marked using rhombus-shaped points. It was shown that, regardless of the nature of changes of liquid penetration into the needle, the bubble growth time fluctuated slightly. The greatest fluctuation of bubble growth time was observed for air volume flow rate in the left needle *q*_*l*_ = 0.0127 l/min. The hydrodynamic interaction affects the waiting time (Fig. 9). Only for the air volume flow rate *q*_*l*_ = 0.0334 l/min, when the frequency of liquid penetration for subsequent cycles of bubble departure is constant, does the waiting time fluctuate slightly. The mean values of bubble growth time, waiting time and their percentage changes vs. air volume flow rate are shown in Fig. 10.

In Fig. 10a, the mean values of bubble growth time (marked using asterisks) and waiting time (rhombus-shaped points) are shown. Bubble growth time and waiting time were calculated for 20 bubble departure cycles in a time series, and the mean values were estimated based on those samples. In Fig. 10b, the percentage changes of bubble growth time and waiting time are shown. The value of percentage changes was calculated as:

2 $${t}_{\%}=\frac{{t}_{max}}{{t}_{min}}*100\%$$

where: *t*_*max*_ –maximum time of bubble growth or waiting time in the analysed time series, *t*_*min*_ –minimum time of bubble growth or waiting time in the analysed time series.

The perturbation of hydrodynamics (caused by changes in the frequency of bubbles departing from the left needle) is the least affected by bubble growth time (Fig. 10a,b) but those changes significantly modify the waiting time. Depending on the nature of the change in the waiting time, there are different scenarios of liquid penetration into the needle and bubble departures. In Fig. 9b, the air volume flow rates (*q*_*l*_ = 0.00827 l/min and *q*_*l*_ = 0.0334 l/min) for which the depth of liquid penetration was similar in all analysed time series (Fig. 6c) are shown above the vertical axis, marked using a filled square. Results obtained showed that the fluctuations in waiting time were close to 2.3% and were lower than fluctuations in bubble growth time (close to 4.6%). In the case of liquid penetration with two repeated depths (*q*_*l*_ = 0.0127 l/min and *q*_*l*_ = 0.0188 l/min—marked with a filled circle above the vertical axis), it was found that the percentage changes of waiting time were greater than the changes of bubble growth time, but those differences are not significant. For *q*_*l*_ = 0.0127 l/min, the changes in waiting time were close to 24.3% and bubble growth time 20.2%. For *q*_*l*_ = 0.0188 l/min, the changes in waiting time were close to 7.4% and bubble growth time 4.9%. In this case, the depths of liquid penetration are not repeatable, or there are periods of non-repeating depths of liquid penetration (Fig. 6a,d), the percentage changes in waiting time were definitely greater than changes in bubble growth time. The changes in waiting time were even close to 42.7% and bubble growth time was 14.4%.

It can be concluded that the hydrodynamic interaction has the greatest impact on the bubble waiting time, i.e. on the processes occurring in the gas supply system of the needle. Synchronization of bubble departures from twin neighbouring needles, occurring due to hydrodynamic interaction, is possible by modifying the waiting time. Moreover, modification of hydrodynamic interaction between bubbles, the bubbles themselves, and gas supply systems can be used to control the bubble departure process.

## Conclusions

In the present paper, the hydrodynamic interactions between bubbles and gas supply systems were experimentally investigated. During the experimental investigation, in one of the needles, the air volume flow rate was constant, but in the neighbouring needle, it was variable. Despite setting a constant air flow rate supply to the needle, the frequency of bubble departures fluctuated, with a change in the flow rate in the neighbouring needle. Fluctuations in bubble departure frequency were associated with the interaction between the bubbles and the gas supply system.

Moreover, in the present paper, it was shown that the hydrodynamic interaction becomes stronger with the increase in air volume flow rate supply to the needle. The occurrence of hydrodynamic interaction modifies bubble growth time slightly, but it significantly modifies the bubble waiting time. It means that the hydrodynamic interaction modifies the process occurring in the gas supply system and consequently the depth of liquid penetration into the needle. It can be shown that synchronized or alternative bubble departures from twin neighboring needles, occurring due to hydrodynamic interaction, is possible by modifying the bubble waiting time but slightly by bubble growth time. Moreover, modification of hydrodynamic interaction between bubbles and the gas supply system can be used to control the bubble departure process from multiple needles.

In the case when the liquid penetration into the needle is repeatable, then the percentage disturbances in bubble growth time and bubble waiting time are close to each other. The process of bubble departures tends to self-organize through modification of the frequency of bubble departure, and this can lead to repeatable liquid movements into the needle with one or two characteristic depths in subsequent cycles of bubble departures.

## Data Availability

Data will be made available on request. Correspondence and requests for materials should be addressed to P. Dzienis.
